# The relationship of peak ankle dorsiflexion angle with lower extremity biomechanics during walking

**DOI:** 10.1002/jfa2.12027

**Published:** 2024-05-29

**Authors:** Tianyu Gao, Zhengye Ma, Nan Yang, Si Zhang, Haitao Shi, Hua Zhang, Shuang Ren, Hongshi Huang

**Affiliations:** ^1^ Department of Sports Medicine Peking University Third Hospital Institute of Sports Medicine of Peking University Beijing Key Laboratory of Sports Injuries Engineering Research Center of Sports Trauma Treatment Technology and Devices Ministry of Education Beijing China; ^2^ Tianjin Key Laboratory of Exercise Physiology and Sports Medicine Institute of Sport, Exercise & Health Tianjin University of Sport Tianjin China; ^3^ Research Center of Clinical Epidemiology Peking University Third Hospital Beijing China

**Keywords:** biomechanics, limited ankle dorsiflexion, pelvic movement, walking

## Abstract

**Purpose:**

Abnormal lower limb movement patterns have been observed during walking in individuals with limited ankle dorsiflexion. The purpose of this study was to investigate the relationships of peak ankle dorsiflexion angle during the stance phase of walking with the lower extremity biomechanics at the corresponding moment and to determine a cutoff value of functional limited ankle dorsiflexion during walking.

**Methods:**

Kinematic and kinetic data of 70 healthy participants were measured during walking. Spearman's correlation coefficients were calculated to establish the association between peak ankle dorsiflexion and angle and moment of ankle, knee, and hip, ground reaction force, and pelvic movement at peak ankle dorsiflexion. All variables significantly related to peak ankle dorsiflexion were extracted as a common factor by factor analysis. Maximally selected Wilcoxon statistic was used to perform a cutoff value analysis.

**Results:**

Peak ankle dorsiflexion positively correlated with ankle plantar flexion moment (*r* = 0.432; *p* = 0.001), ankle external rotation moment (*r* = 0.251; *p* = 0.036), hip extension angle (*r* = 0.281; *p* = 0.018), hip flexion moment (*r* = 0.341; *p* = 0.004), pelvic ipsilateral rotation angle (*r* = 0.284; *p* = 0.017), and medial, anterior, and vertical ground reaction force (*r* = 0.324; *p* = 0.006, *r* = 0.543; *p* = 0.001, *r* = 0.322; *p* = 0.007), negatively correlated with knee external rotation angle (*r* = −0.394; *p* = 0.001) and hip adduction angle (*r* = −0.256; *p* = 0.032). The cutoff baseline value for all 70 participants was 9.03°.

**Conclusions:**

There is a correlation between the peak ankle dorsiflexion angle and the lower extremity biomechanics during walking. If the peak ankle dorsiflexion angle is less than 9.03°, the lower limb movement pattern will change significantly.

## 1 INTRODUCTION

Triceps surae tension and insufficient talar slip may lead to limited ankle dorsiflexion [1]. Limited ankle dorsiflexion increases the risk of foot and ankle disorders such as Achilles tendinopathy, chronic ankle instability, plantar fasciitis, and metatarsal stress fracture [2, 3, 4, 5]. Individuals with limited ankle dorsiflexion may compensate by increasing the activity of other joints in the lower extremity kinetic chain, such as tibial internal rotation, knee valgus, hip adduction and internal rotation, or pelvic tilt [6, 7, 8, 9], making them more susceptible to knee disorders, such as patellofemoral pain and anterior cruciate ligament injuries [10, 11]. During the forefoot support phase of the gait cycle, the tibia and foot move forward around the forefoot axis relative to the foot to peak ankle dorsiflexion [12]. It is necessary to clarify the effect of limited ankle dorsiflexion during walking on the entire lower extremity kinetic chain.

Previous studies have shown that limited ankle dorsiflexion leads to gait changes. Aquino et al. found that individuals with passive measures of limited ankle dorsiflexion had shorter stride lengths, lower peak ipsilateral pelvic rotation angles, lower peak hip, and knee flexion angles during the stance phase, and lower peak ankle and forefoot dorsiflexion angles during walking [13]. However, the range of ankle dorsiflexion during gait does not correlate with the maximum angle measured passively, and underutilization of the ankle dorsiflexion angle can also lead to abnormal movement patterns [14, 15]. Ota S et al. reported that knee kinematics and kinetics in the sagittal and frontal planes were affected by limited ankle dorsiflexion, with a significant increase in peak knee extension angle and knee varus moment during terminal stance [16]. A peak ankle dorsiflexion angle of approximately 10° is required during the stance phase of walking, and insufficient ankle dorsiflexion is indicative of functional limitation [17]. Our preliminary study found that during walking the ankle plantar flexion moment, hip extension angle, medial ground reaction force, anterior ground reaction force, and pelvis ipsilateral drop in the restricted group (peak ankle dorsiflexion angle less than 10°) were lower, and the knee external rotation angle was greater [18]. However, the relationship of peak ankle dorsiflexion angle with lower extremity biomechanics is unclear, and further research is needed to understand how limited ankle dorsiflexion affects gait.

For this reason, this study aimed (1) to investigate the correlation between the peak ankle dorsiflexion angle during the stance phase of walking and the angles and moments of the ankle, knee, hip, ground reaction forces, and pelvic movements at the corresponding moment, and (2) to calculate a cutoff value for the peak ankle dorsiflexion angle that accurately assesses functional limited ankle dorsiflexion during walking. We hypothesized that functional limited ankle dorsiflexion would be associated with reduced lower extremity activity in the sagittal plane and increased compensatory activity in the frontal and transverse planes.

## 2 MATERIALS AND METHODS

### 2.1 Participants

This cross‐sectional study included 70 healthy participants, including 63 males and 7 females, with an average age of 28.99 years (Table 1). This study was approved by Peking University Third Hospital Medical Science Research Ethics Committee, and all participants read and signed an approved informed consent document before the experiment (M2023360). We have conducted a step of preliminary study, which this study builds on to explore further [18]. Some of the participants in the preliminary study were identical to those in the present study. The same inclusion criteria were applied to the additional participants: (1) normal body mass index (18.5–24.9); (2) no cognitive disorder or nervous system diseases; (3) no injuries or diseases of lower limbs or pelvis that lead to abnormal gait; (4) no surgical history of lower limbs or pelvis. None of the participants in this study reported adverse events, and no pain or discomfort was reported during data collection. We used G*Power software (version 3.1.9.6) to calculate the sample size [19], which showed that a minimum of 42 participants were required for this correlation study, given that the correlations were more than moderate (*r* = 0.5, *α* = 0.05, *β* = 0.05).

**TABLE 1:** The demographic data of the participants.

Variables	Mean (SD)
Male/female (*n*)	63/7 (70)
Age (years)	28.99 (9.84)
Height (cm)	172.81 (7.80)
Mass (kg)	73.10 (12.71)

### 2.2 Data collection

The methods used to collect the data were consistent with our preliminary study [18]. During biomechanical testing, reflective markers were placed bilaterally at acromions, anterior superior iliac spines, posterior superior iliac spines, lateral thighs, lateral femoral condyles, tibial tuberosities, anterior superior shanks, anterior inferior shanks, lateral legs, lateral malleoli, heels, first, second and fifth metatarsophalangeal joints, medial femoral condyles, and medial malleoli. An 8‐camera high‐speed infrared motion capture system (Vicon, UK) with a sampling rate of 100 Hz was used to collect static and dynamic three‐dimensional motion information from the subject. Two embedded force plates (AMTI, USA) were used to collect the ground reaction forces at a sample rate of 1000 Hz. A synchronization box (AMTI, GEN 5) was used to synchronize the high‐speed motion capture system with the force plates to collect kinematic and kinetic data during the test. All subjects were asked to walk from a predetermined point so that one foot inadvertently stepped on the first force plate and the other on the second force plate. They walked at least seven steps, with the feet landing on force plates on the third and fourth steps. A successful trial was defined as each foot making contact with the force plates at a self‐selected speed. The data collection was completed after recording five successful gait trials.

### 2.3 Data processing

The 3D coordinates of all marker points collected in this study were smoothed using Butterworth low‐pass filtering with a cutoff frequency of 12 Hz. A Butterworth low‐pass filter with a cutoff frequency of 100 Hz was also used to smooth the ground reaction forces. Time‐series data for the kinematics and kinetics variables were calculated using Visual 3D software (C‐motion, USA) by inverse dynamics. Biomechanical modeling of rigid bodies using static tests with the talonavicular joint in the neutral position. The moments were expressed as internal moments. Moments were normalized by height and weight, and ground reaction forces were normalized by the product of weight and gravity acceleration. One side of the lower limbs were randomly selected for statistical analysis.

### 2.4 Statistical analysis

Descriptive statistics were used to summarize the demographic variables and biomechanical variables, mean and standard deviation or median and interquartile range were used to describe the results. A correlation analysis (Spearman rho correlation coefficient) was used to investigate the association between the independent variable (peak ankle dorsiflexion) and each dependent variable (angle and moment of ankle, knee and hip, ground reaction force and pelvic movement at peak ankle dorsiflexion).

All the dependent variables significantly related to peak ankle dorsiflexion were extracted as a common factor by factor analysis. The association between peak ankle dorsiflexion and common factor as a continuous variable was analyzed. Continuous peak ankle dorsiflexion was dichotomized for ease of clinical utility. A cutoff point analysis, in which selected values of peak ankle dorsiflexion were examined as candidates for the cutoff point, was performed using the maximally selected Wilcoxon statistic. Rather than arbitrarily choosing the median, the cutoff value was chosen according to a maximum relative risk and a minimum *p* value. Correlation analysis and factor analysis were performed using SPSS software (version 26.0), maximally selected Wilcoxon statistic was performed using R software (version 4.3.0), and the 2‐sided *p*‐values <0.05 were considered statistically significant.

## 3 RESULTS

**FIGURE 1:**
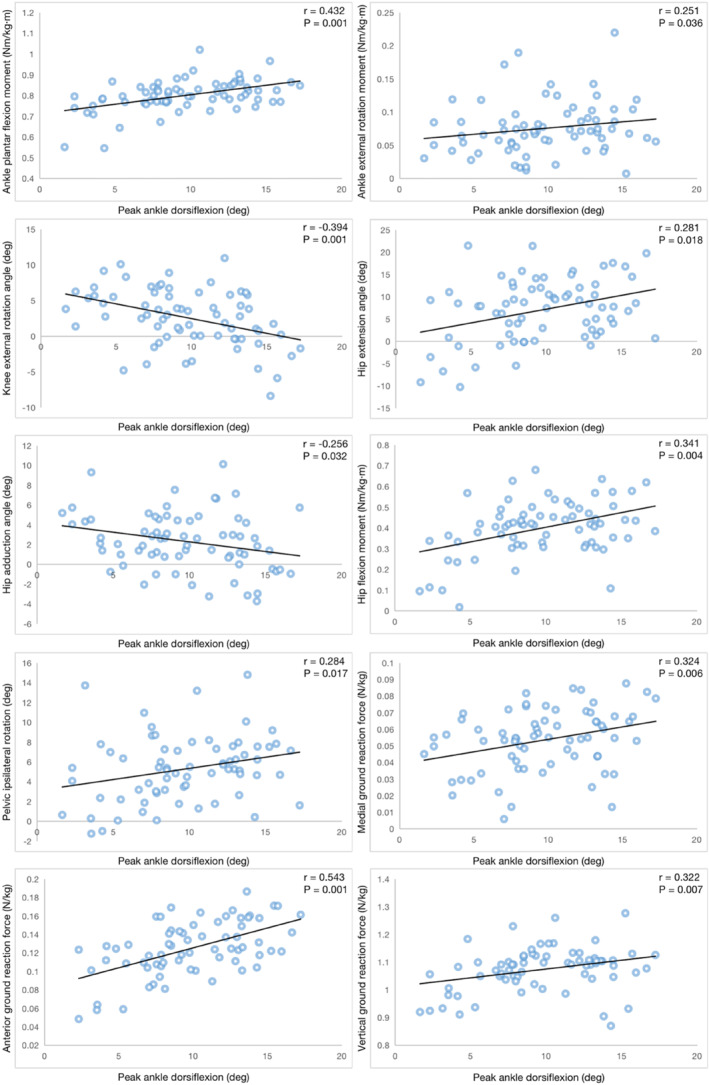
Scatter plot for the association between peak ankle dorsiflexion and the biomechanical variables during walking.

Descriptive results regarding demographic data and biomechanical data at peak ankle dorsiflexion are presented in Table 1 and Table 2. Peak ankle dorsiflexion positively correlated with ankle plantar flexion moment (*r* = 0.432; *p* < 0.01), ankle external rotation moment (*r* = 0.251; *p* < 0.05), hip extension angle (*r* = 0.281; *p* < 0.05), hip flexion moment (*r* = 0.341; *p* < 0.01), pelvic ipsilateral rotation angle (*r* = 0.284; *p* < 0.05), medial ground reaction force (*r* = 0.324; *p* < 0.01), anterior ground reaction force (*r* = 0.543; *p* < 0.01), and vertical ground reaction force (*r* = 0.322; *p* < 0.01), negatively correlated with knee external rotation angle (*r* = −0.394; *p* < 0.01) and hip adduction angle (*r* = −0.256; *p* < 0.05). No correlations were found between peak ankle dorsiflexion and other biomechanical variables (Table 3). The significant correlations were shown in Figure 1.

**TABLE 2:** The biomechanical data at peak ankle dorsiflexion of the participants.

Variables	Median (IQR)
Ankle	
Ankle dorsiflexion angle (deg)	9.47 (5.83)
Ankle valgus angle (deg)	2.85 (6.41)
Ankle internal rotation angle (deg)	0.61 (4.15)
Ankle plantar flexion moment (Nm/kg·m)	0.80 (0.08)
Ankle varus moment (Nm/kg·m)	0.06 (0.10)
Ankle external rotation moment (Nm/kg·m)	0.07 (0.04)
Knee	
Knee flexion angle (deg)	6.58 (5.78)
Knee abduction angle (deg)	0.52 (3.84)
Knee external rotation angle (deg)	2.89 (5.77)
Knee flexion moment (Nm/kg·m)	0.08 (0.13)
Knee abduction moment (Nm/kg·m)	0.16 (0.09)
Knee external rotation moment (Nm/kg·m)	0.05 (0.05)
Hip	
Hip extension angle (deg)	7.90 (10.52)
Hip adduction angle (deg)	2.36 (3.72)
Hip external rotation angle (deg)	0.04 (9.41)
Hip flexion moment (Nm/kg·m)	0.42 (0.15)
Hip abduction moment (Nm/kg·m)	0.41 (0.13)
Hip external rotation moment (Nm/kg·m)	0.02 (0.06)
Pelvis	
Pelvis anterior tilt (deg)	9.22 (6.44)
Pelvis ipsilateral drop (deg)	1.21 (2.28)
Pelvic ipsilateral rotation (deg)	5.27 (4.57)
Ground reaction force	
Medial ground reaction force (N/kg)	0.05 (0.02)
Anterior ground reaction force (N/kg)	0.12 (0.04)
Vertical ground reaction force (N/kg)	1.09 (0.08)

**TABLE 3:** Spearman correlation coefficients (*r*) between peak ankle dorsiflexion and the biomechanical variables at peak ankle dorsiflexion.

	Peak ankle dorsiflexion	
	*r*	*p*
Ankle		
Ankle dorsiflexion angle	1	/
Ankle valgus angle	−0.037	0.758
Ankle internal rotation angle	0.149	0.218
Ankle plantar flexion moment	0.432	0.001**
Ankle varus moment	−0.139	0.250
Ankle external rotation moment	0.251	0.036*
Knee		
Knee flexion angle	−0.002	0.984
Knee abduction angle	0.154	0.204
Knee external rotation angle	−0.394	0.001**
Knee flexion moment	−0.189	0.118
Knee abduction moment	−0.010	0.934
Knee external rotation moment	0.221	0.066
Hip		
Hip extension angle	0.281	0.018*
Hip adduction angle	−0.256	0.032*
Hip external rotation angle	0.184	0.128
Hip flexion moment	0.341	0.004**
Hip abduction moment	0.061	0.616
Hip external rotation moment	−0.154	0.204
Pelvis		
Pelvis anterior tilt	−0.167	0.168
Pelvis ipsilateral drop	0.184	0.128
Pelvic ipsilateral rotation	0.284	0.017*
Ground reaction force		
Medial ground reaction force	0.324	0.006**
Anterior ground reaction force	0.543	0.001**
Vertical ground reaction force	0.322	0.007*

**FIGURE 2:**
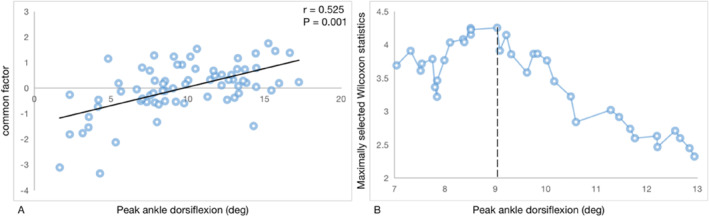
(A) Scatter plot for the association between peak ankle dorsiflexion and the common factor. (B) The cutoff value of peak ankle dorsiflexion was 9.03°, which was defined by using the maximally selected Wilcoxon statistic.

Factor analysis was performed on 10 biomechanical variables significantly related to peak ankle dorsiflexion (KMO = 0.728, *p* < 0.01). The first common factor was extracted as a new variable that explained 38.37% data variation. Correlation analysis showed that peak ankle dorsiflexion was significantly correlated with the common factor (*r* = 0.525, *p* < 0.01) (Figure 2). In order to determine the biomechanical consequence of baseline peak ankle dorsiflexion, according to the cutoff point by the maximally selected Wilcoxon statistic, participants were divided into two classes with respect to peak ankle dorsiflexion. The cutoff baseline value for all 70 participants was 9.03° (Figure 2). Therefore, functional limited ankle dorsiflexion was defined as peak ankle dorsiflexion <9.03° during the stance phase of walking.

## 4 DISCUSSION

To the best of our knowledge, this study was the first to correlate the peak ankle dorsiflexion during the stance phase of walking with the lower extremity biomechanics at the corresponding moment. Most previous studies assessed whether limited ankle dorsiflexion by passive measures of range of motion [20]. The inability to fully utilize the ankle dorsiflexion range of motion during exercise was referred to as functional limited ankle dorsiflexion, which may also be associated with an abnormal movement pattern [14, 15, 17]. The aim of this study was to investigate the effects of functional limited ankle dorsiflexion during walking on lower extremity biomechanics using correlation analysis. We hypothesized that the lower extremities would have reduced activity in the sagittal plane and increased compensatory activity in the frontal and transverse planes when the peak ankle dorsiflexion angle was low. The results of the study basically supported our hypothesis that limited peak ankle dorsiflexion angle was significantly correlated with lower ankle plantarflexion moment, ankle external rotation moment, hip extension angle and hip flexion moment, and greater knee external rotation angle and hip adduction angle. In addition, limited peak ankle dorsiflexion angle was significantly correlated with lower ipsilateral pelvic rotation, medial ground reaction forces, anterior ground reaction forces, and vertical ground reaction forces. Finally, we innovatively proposed that a peak ankle dorsiflexion angle of 9.03° could be used as a cutoff to diagnose functional ankle dorsiflexion limitation during walking.

During the terminal stance phase, the Achilles tendon was stretched at the peak ankle dorsiflexion, the Achilles tendon began to release its elastic potential energy and began to contract, causing ankle plantarflexion, during which the ankle plantarflexion moment was the main driving force that pushed the lower extremity into the swing phase [21, 22]. It has been suggested that during push‐off, the plantarflexors provide critical stabilization of the knee while also providing propulsion [23]. It has been demonstrated that when the ankle plantarflexion moment is insufficient, balance and walking speed may be reduced [24]. Our results showed that when the peak ankle dorsiflexion angle was lower, the ankle plantarflexion moment at the corresponding moment was lower. This means individuals with limited ankle dorsiflexion may experience reduced ankle stability and slower walking speeds. Ankle instability may lead to a compensatory pattern throughout the lower extremity and increase the risk of injury [25]. Reduced walking speed was one of the factors that increased the risk of developing knee osteoarthritis [26]. In addition, our results showed that the ankle external rotation moment was lower at the moment corresponding to the peak ankle dorsiflexion. The externally rotated and abducted position of the ankle during the stance phase may be responsible for the simultaneous generation of an ankle external rotation moment to provide propulsion to the lower extremity during push‐off [27].

The results of the correlation analysis indicate a negative correlation between the peak ankle dorsiflexion angle and the knee external rotation angle at the corresponding moment. Based on the motor chain coupling of the lower limb, it is possible to explain that a lack of ankle activity in the sagittal plane may result in compensation by the knee in the transverse plane with excessive external rotation. During the stance phase, the knee is in a slightly externally rotated position at initial landing, gradually internally rotates at the beginning of the loading response phase, peaks in internal rotation at the end of the loading response phase, and then the knee begins to gradually externally rotate [28]. At the terminal stance, the heel lifts and the knee starts to flex, allowing the femur to move forward in preparation for the swing of the lower limb; those with limited ankle dorsiflexion can be assisted in this maneuver by increasing the knee external rotation angle. Increased knee external rotation angles result in greater activation of the lateral quadriceps, which subsequently alters patellofemoral joint loading, which may lead to the development of patellofemoral pain [29]. Additionally, several studies have indicated that an excessive knee external rotation is associated with the mechanism of anterior cruciate ligament injury [30]. Individuals with limited ankle dorsiflexion have compensatory activities at the knee that make them more susceptible to knee disease or injury.

Peak ankle dorsiflexion was positively correlated with hip extension angle and hip flexion moment at the corresponding moment, and negatively correlated with hip adduction angle. As the body's center of gravity moves forward over the center of the hip joint, the hip extends and simultaneously generates a moment of flexion. This process causes an adduction angle of the hip due to the contralateral pelvic drop. Lower hip extension angles and hip flexion moments lead to higher loads in the hip joint, which are associated with the development of hip osteoarthritis [31, 32]. Greater hip abduction angle is associated with increased iliotibial band hardness, which may lead to greater friction in the underlying lateral femoral epicondyle and thus lead to iliotibial band syndrome [33, 34]. In addition, increased hip adduction angle appears to be associated with knee injuries. The increase of hip adduction angle will make the quadriceps muscle line move outward, which will increase the pressure of the patellofemoral joint and lead to patellofemoral pain [35]. Due to alterations in the force line of the lower limbs, it may result in patellar instability, increasing the risk of patella dislocation [36]. Therefore, individuals with limited ankle dorsiflexion may be more prone to hip or knee pain.

Consistent with previous research [13], our research results showed that the lower peak ankle dorsiflexion angle, the lower ipsilateral pelvic rotation. The pelvis is the axial bone that connects the spine to the lower limbs and is connected to the femur by the hip joint, so when the hip is extended, the pelvis begins to rotate on the same side. So reduced ipsilateral pelvis rotation may lead to injuries to the hip joint in the same way as reduced hip extension. As the pelvis is connected to the spine, changes in pelvic kinematics may also be associated with lumbosacral injuries. A lower pelvic rotation angle leads to lumbopelvic region stiffness during walking, which is associated with the onset and progression of low back pain [37]. In addition, restricted pelvic rotation during walking also significantly reduces stride and step length, as well as gait speed [38]. In other words, the reduced ankle dorsiflexion may have compromised the forward progression of the body during gait, and may have a negative effect on the lumbopelvic region and the hip.

Ground reaction forces are related with limb kinematics during walking and may reflect changes in lower limb strength during the stance phase. Previous studies have shown an association between reduced ground reaction forces and changes in gait parameters [39]. At peak ankle dorsiflexion, the ankle plantarflexor muscle groups forcefully pedal off the ground, and the ground reaction force at the corresponding moment responds to the magnitude of the lower limb pedal force. Peak ankle dorsiflexion was positively correlated with medial, anterior and vertical ground reaction forces at the corresponding moment. If the ankle dorsiflexion is insufficient, the corresponding ground reaction force would decrease, resulting in insufficient forward propulsion. The results of our study provided an explanation for the shortened step length in individuals with limited ankle dorsiflexion [13].

Overall, functional limited ankle dorsiflexion during walking affected all lower extremity joints and the pelvis. In terms of kinematics, there was an increase in knee external rotation angle and hip adduction angle and a decrease in hip extension angle and pelvic ipsilateral rotation; in terms of kinetics, there was a decrease in ankle plantarflexion moment, ankle external rotation moment, hip flexion moment and medial, anterior and vertical ground reaction forces. Functional limited ankle dorsiflexion was associated with decreased lower extremity activity in the sagittal plane and increased activity in the frontal and transverse planes appearing as compensatory activity, along with decreased forward propulsion of the lower extremity. Functional limited ankle dorsiflexion resulting in biomechanical changes may lead to a range of adverse effects including impaired balance, reduced step speed [24] and shortened stride length [13], as well as knee osteoarthritis [26], patellofemoral pain [29, 35], iliotibial band syndrome [33, 34], hip osteoarthritis [31, 32], and low back pain [37]. However, how to assess functional limited ankle dorsiflexion is a challenge. In this study, statistical methods were used to calculate a cutoff point to aid assessment and rehabilitation. Factor analysis was first used to extract the principal components of the biomechanical parameters that were significantly correlated with peak ankle dorsiflexion angle. The extracted common factor represents the lower limb movement pattern related to the ankle dorsiflexion angle. Then maximally selected test statistics was performed on peak ankle dorsiflexion angle and the extracted common factor. The results of the maximally selected test statistics showed that the most significant difference was when the cutoff point was 9.03°. It is suggested that there is the most significant difference in lower limb movement patterns between the two groups when they are grouped at 9.03°. Therefore, 9.03° can be used as a criterion for assessing functional limited ankle dorsiflexion, and the lower limb movement pattern will change significantly when the peak ankle dorsiflexion angle is less than 9.03° during walking.

There are some limitations in this study. Firstly, we did not include participants of the same gender, so we do not know whether gender would have seriously affected the results of the study. In addition, our results only apply to the walking task, and the effects of insufficient ankle dorsiflexion in other tasks need to be further investigated.

## 5 CONCLUSIONS

Limited peak ankle dorsiflexion during walking was associated with an increase in knee external rotation angle and hip adduction angle at the corresponding moment, and with a decrease in ankle plantarflexion moment, ankle external rotation moment, hip extension angle, hip flexion moment, ipsilateral pelvis rotation, and medial, anterior, and vertical ground reaction forces. The cutoff value of diagnosing functional limited ankle dorsiflexion during walking can be set at 9.03°. This study elucidates the relationship of peak ankle dorsiflexion angle with lower extremity biomechanics during walking and provides diagnostic criteria for functional limited ankle dorsiflexion to help with clinical rehabilitation program development.

## AUTHOR CONTRIBUTIONS

**Tianyu Gao**: Conceptualization; data curation; methodology; writing ‐ original draft preparation. **Zhengye Ma**: Investigation; methodology; resources. **Nan Yang**: Resources. **Si Zhang**: Resources. **Haitao Shi**: Resources. **Hua Zhang**: Formal Analysis. **Shuang Ren**: Writing – review & editing. **Hongshi Huang**: Supervision; writing – review & editing.

## CONFLICT OF INTEREST STATEMENT

The authors declare that the research was conducted in the absence of any commercial or financial relationships that could be construed as a potential conflict of interest.

## ETHICS STATEMENT

Ethics approval was obtained from Peking University Third Hospital Medical Science Research Ethics Committee (M2023360).

## CONSENT TO PARTICIPATE

Informed consent was obtained from all individual participants included in the study.

## CONSENT TO PUBLISH

All authors have approved the release of their work to the public.

## Data Availability

Research data are not shared.
